# Open inguinal hernia repair under combined transversalis fascia plane and transversus abdominis plane blocks in a high-risk cardiac patient

**DOI:** 10.1007/s10029-026-03628-4

**Published:** 2026-03-31

**Authors:** Ilke Tamdogan

**Affiliations:** Department of Anesthesiology and Reanimation, Faculty of Medicine Giresun, Giresun University, Giresun, Turkey

**Keywords:** Inguinal hernia, Heart failure, Pacemaker, Nerve block, Ultrasound-guided anesthesia

## Abstract

**Background:**

Selecting an optimal anesthetic technique for inguinal hernia repair is particularly challenging in patients with severe cardiac comorbidities and ongoing anticoagulant therapy. General anesthesia may increase cardiopulmonary instability, whereas neuraxial techniques carry risks of hypotension and bleeding complications.

**Case presentation:**

We report the case of a 76-year-old man with advanced heart failure (ejection fraction 20%), New York Heart Association (NYHA) class III symptoms, pacemaker dependency, and chronic warfarin therapy who underwent elective open right inguinal hernia repair. Ultrasound-guided unilateral transversalis fascia plane (TFP) block (30 mL of 0.25% bupivacaine) combined with a transversus abdominis plane (TAP) block (20 mL of 0.25% bupivacaine) was performed as the sole anesthetic technique. Adequate sensory blockade over T12–L1 dermatomes was achieved within 20 minutes. Surgery using the Lichtenstein technique was completed in 45 minutes with stable hemodynamic parameters, without the need for conversion to general anesthesia, surgical infiltration, or intraoperative opioid supplementation.

**Results:**

Postoperative pain was well controlled with multimodal non-opioid analgesia, with numerical rating scale (NRS) scores ≤2 during the first 24 hours. No rescue opioid analgesia was required. The patient had an uneventful recovery and was discharged on postoperative day 2.

**Conclusion:**

The combination of transversalis fascia plane and TAP blocks may represent a feasible and effective anesthetic alternative for open inguinal hernia repair in carefully selected high-risk cardiac patients in whom general or neuraxial anesthesia is undesirable.

## Open inguinal hernia repair under combined transversalis fascia plane and transversus abdominis plane blocks in a high-risk cardiac patient

Inguinal hernia repair is one of the most frequently performed elective surgical procedures globally, with the patient demographic often characterized by advanced age and multiple cardiopulmonary and metabolic comorbidities. In this context, the selection of anesthetic technique is a crucial determinant of perioperative morbidity and mortality, particularly in frail patients and those undergoing anticoagulant or antiplatelet therapy. Given that general or neuraxial anesthesia may elevate the risk of hemodynamic instability, myocardial ischemia, respiratory depression, and neuraxial hematoma in high-risk cases, there is increasing interest in regional approaches that provide more limited systemic effects and enhanced hemodynamic tolerability.

Ultrasound-guided abdominal wall fascial plane blocks, including the transversus abdominis plane (TAP) block, transversalis fascia plane block (TFPB) and quadratus lumborum (QL) block have become integral to multimodal analgesia for inguinal hernia repair and can reduce postoperative pain and opioid consumption by variably affecting the lower thoracic, upper lumbar segments and the ilioinguinal/iliohypogastric nerves, depending on injection technique and local anesthetic spread [1–3]. However, given the complex innervation of the inguinal region and the substantial contribution of L1-level nerves to surgical pain, these blocks are predominantly described as adjunctive analgesic techniques to general or neuraxial anesthesia, and evidence supporting their use as sole techniques for surgical anesthesia remains limited to a small number of case reports and case series [4, 5]. In this report, we present a case in which open inguinal hernia repair was successfully performed without the need for general anesthesia using an ultrasound-guided combination of transversalis fascia plane and transversus abdominis plane blocks in a patient who was not suitable for neuraxial anesthesia due to advanced cardiac comorbidities.

A 76-year-old, 80-kg male patient (American Society of Anesthesiologists physical status III) was scheduled for elective open right inguinal hernia repair. His medical history was notable for advanced heart failure with severe left ventricular systolic dysfunction. Transthoracic echocardiography performed in August 2025 demonstrated a markedly reduced left ventricular ejection fraction of 20%, global hypokinesia, and left atrial enlargement (LA diameter: 33 mm). Pacemaker leads were visualized within the right heart chambers. The patient had significant functional limitation consistent with New York Heart Association (NYHA) class III symptoms. Additional comorbidities included coronary artery disease, hypertension, and diabetes mellitus. He had previously undergone surgical aortic valve replacement for an ascending aortic aneurysm. A dual-chamber permanent pacemaker was implanted in 2022 for complete atrioventricular block.

The patient was receiving chronic oral anticoagulation with warfarin. For perioperative anticoagulation management, warfarin was discontinued 7 days before surgery, and bridging anticoagulation with enoxaparin 40 mg subcutaneously twice daily was initiated; the last dose was withheld 12 h preoperatively. On arrival to the operating room, the international normalized ratio (INR) was 1.4. Given the patient’s severe cardiac dysfunction, device dependency, and anticoagulation status, the anesthetic strategy prioritized avoidance of general and neuraxial anesthesia, and the procedure was planned under peripheral regional anesthesia.

In the operating room, standard monitoring, including ECG, noninvasive blood pressure, and pulse oximetry was applied. Premedication consisted of midazolam 2 mg and fentanyl 50 µg intravenously. Supplemental oxygen was administered via a simple face mask at 3–4 L/min.

**Fig. 1:**
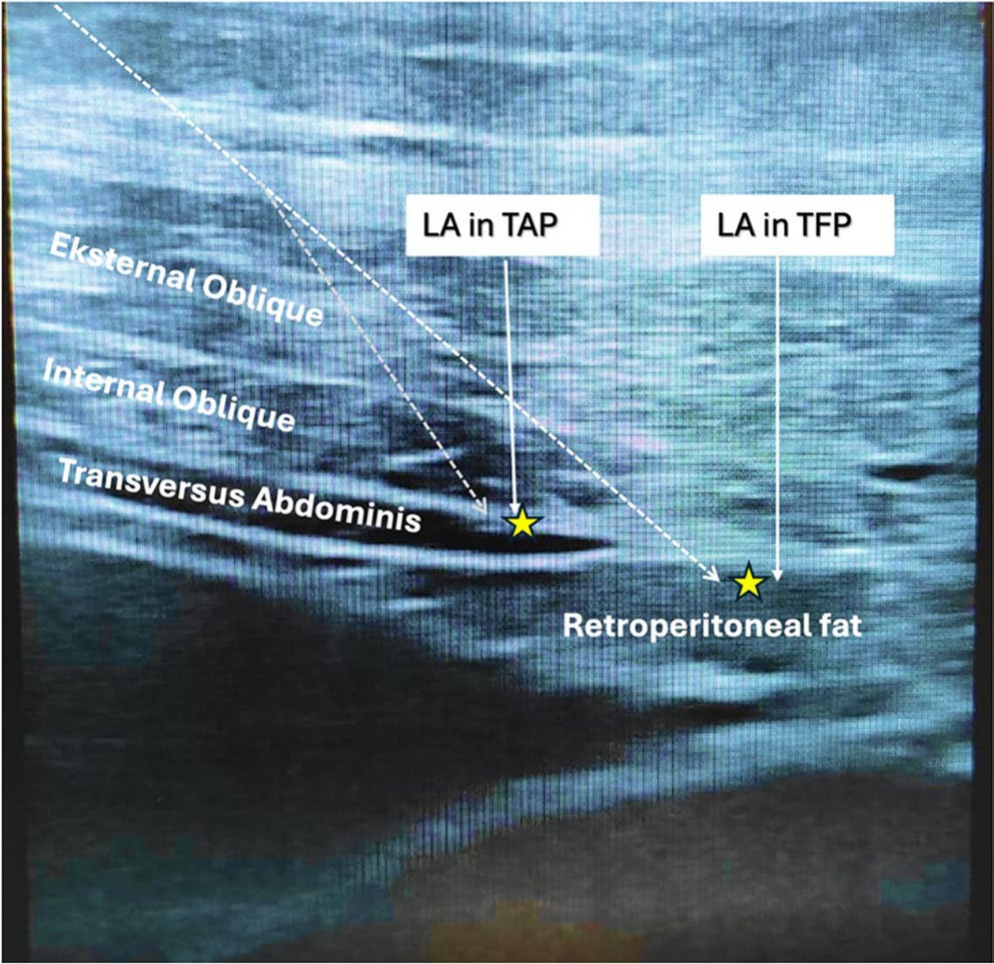
Ultrasound image demonstrating combined unilateral transversalis fascia plane (TFP) and transversus abdominis plane (TAP) blocks. The abdominal wall muscle layers (external oblique, internal oblique, and transversus abdominis) are identified. The in-plane needle trajectory is illustrated. Local anesthetic spread between the internal oblique and transversus abdominis muscles is labeled as “LA in TAP.” Local anesthetic spread beneath the transversus abdominis muscle is labeled as “LA in TFP,” with characteristic displacement of retroperitoneal fat, confirming correct access to the transversalis fascia plane

With the patient in the supine position, a linear ultrasound probe (6–13 MHz) was positioned transversely cranial to the iliac crest. Utilizing an in-plane approach, a 100-mm block needle was advanced through the skin, subcutaneous tissue, and the external and internal oblique muscles toward the termination of the transversus abdominis muscle, with the transversalis fascia visualized beneath it. Following confirmation of the correct plane by hydrodissection, 30 mL of 0.25% bupivacaine was injected into the transversalis fascia plane, with displacement of retroperitoneal fat observed during injection. The needle was subsequently withdrawn and redirected between the internal oblique and transversus abdominis muscles, where 20 mL of 0.25% bupivacaine was administered to perform a unilateral transversus abdominis plane (TAP) block (Fig. 1). Within approximately 20 min, decreased pinprick sensation was noted over the T12–L1 dermatomes, including the inguinal region, and surgery was initiated via a Lichtenstein incision. Because of pacemaker dependency and the use of electrocautery, an electromagnetic interference (EMI) mitigation strategy was implemented. Bipolar electrocautery was preferred to minimize EMI. Intraoperatively, the pacemaker was programmed to an asynchronous pacing mode; continuous ECG and hemodynamic monitoring was maintained, and external pacing/defibrillation capabilities were immediately available. No additional intraoperative opioids were required, and conversion to general anesthesia was not necessary. The procedure was completed uneventfully in 45 min, and postoperative intensive care admission was not required. During the first 24 postoperative hours, under a standard multimodal analgesic regimen (intravenous paracetamol 1 g every 6 h and dexketoprofen every 12 h), numerical rating scale (NRS) pain scores remained ≤ 2, and no rescue opioids were required. The patient experienced an uncomplicated postoperative course and was discharged on postoperative day 2.

Written informed consent was obtained from the patient for publication of this case.

The sensory innervation of the inguinal region is intricate, involving the ilioinguinal and iliohypogastric nerves (primarily L1) and contributions from the lower thoracic segments, which significantly influence the development of surgical pain. This anatomical complexity accounts for the traditional description of abdominal wall plane blocks as adjunctive techniques to general or neuraxial anesthesia, primarily to enhance postoperative analgesia. Comprehensive reviews of abdominal wall blocks have highlighted that dermatomal coverage and block reliability may vary based on the technique employed, the volume injected, and the continuity of fascial planes [6, 7].

The TFPB has been proposed as a more caudal and anterior approach targeting the T12–L1 nerves, which are clinically significant for inguinal surgery [8, 9]. In contrast, the conventional TAP block may provide variable sensory coverage across the lower thoracic–upper lumbar segments, and its ability to consistently cover the L1 territory (ilioinguinal/iliohypogastric nerves) may be inconsistent in some patients [6, 7]. From this perspective, combining TFPB with a TAP block may offer a complementary strategy: TFPB may enhance the L1-related blockade, while the TAP block may extend the abdominal wall sensory coverage. Case reports and brief communications have described the use of TFPB alone or in combination with TAP as an alternative anesthetic strategy for inguinal hernia repair in medically complex patients [9–11].

In the present case, a unilateral TFPB–TAP combination produced clinically adequate blockade over the T12–L1 dermatomes, allowing surgery to proceed without surgical field infiltration or conversion to general anesthesia. The patient’s severe left ventricular systolic dysfunction (ejection fraction 20%) and NYHA class III symptoms increased concern for hypotension and reduced cardiac reserve with neuraxial sympathectomy, as well as cardiopulmonary stress during induction, airway manipulation, and positive-pressure ventilation under general anesthesia. In addition, although anticoagulation does not universally preclude neuraxial anesthesia, it requires individualized risk–benefit assessment. Warfarin had been discontinued 7 days preoperatively with bridging anticoagulation, and the INR on the day of surgery was 1.4; nonetheless, a peripheral regional strategy was favored to avoid neuraxial puncture in a medically fragile patient. The absence of further intraoperative opioid requirement beyond minimal premedication and low early postoperative pain scores without rescue opioids support that the regional technique provided both intraoperative conditions and early analgesia.

This report has limitations inherent to a single case. Dermatomal assessment is subjective and does not fully characterize block distribution. The approach may not be reliable in all patients given anatomical variability, local anesthetic spread, and surgical factors.

In conclusion, unilateral combination of ultrasound-guided TFPB and TAP block may be a feasible anesthetic alternative for open inguinal hernia repair in carefully selected patients in whom general or neuraxial anesthesia is undesirable due to severe cardiac disease and anticoagulation-related concerns.

## Data Availability

No datasets were generated or analyzed during the current study. Data sharing is not applicable to this article.
